# “Stay at Home”: The Effects of the COVID-19 Lockdown on Household Food Waste in Colombia

**DOI:** 10.3389/fpsyg.2021.764715

**Published:** 2021-10-28

**Authors:** Daniela Mejia, Manuel Diaz, Andres Charry, Karen Enciso, Oscar Ramírez, Stefan Burkart

**Affiliations:** ^1^Independent Researcher, Cali, Colombia; ^2^Alliance Bioversity International and CIAT, Cali, Colombia

**Keywords:** COVID-19, food waste, pandemic, consumer behavior, logistic regression, random forest

## Abstract

Household food waste represents one of the main challenges for sustainable development as this directly affects the economy of food consumers, the loss of natural resources and generates additional greenhouse gas emissions. The COVID-19 pandemic and its mitigation strategies caused one of the most serious economic crises in recent decades and could become the worst economic crisis that Latin America has had in its history. The objective of this study is to analyze changes in food waste behavior during the COVID-19 lockdown in Colombia in 2020, applying the Theory of Planned Behavior (TPB). For this purpose, we conducted a survey with 581 Colombian food consumers, which examined the influence of intentions to not waste food, subjective norms, some situational predictors, questions related to the COVID-19 pandemic, and the control of perceived behavior on food waste. The results suggest that the TPB can predict the intention to not waste food and, through it, the actual household food waste behavior, considering the lockdown in Colombia as an external shock. We observe that regarding the intention to not waste food, the most relevant variables are attitudes, subjective norms, control of the perceived behavior, and concerns regarding the Covid-19 pandemic. These variables increase the probability on average by a 0.8 Odds Ratio that the intention not to waste food increases, too. Regarding food waste behavior, whether it is considered ordinal or nominal, we see that the most relevant variables are intention, financial attitudes, and control of perceived behavior, doubling the probability that food waste behavior will improve. Based on the results, we provide recommendations for interested stakeholders that can help in the design of instruments for household food waste reduction.

## Introduction

Food waste and loss account for about a third of the global annual food production volume (Gustavsson et al., 2011) and, despite the end consumer being the protagonist (Griffin et al., 2009), 13.8% of the global food leakage occurs at many different stages of the food value chains (FAO, 2017). There is a difference between what is considered food loss and food waste. While food loss occurs throughout the value chains, food waste happens at the retail and consumption levels (FAO, 2019). According to FAO (2011), ~54% of the global annual food leakage corresponds to loss, and 46% to waste.

Combatting food waste makes part of the Sustainable Development Goals (SDG), postulated by the United Nations in the 2030 Agenda for Sustainable Development (i.e., UN-SDG 12: Responsible Consumption and Production) (UN., 2015). Within UN-SDG 12, global food loss and waste are considered by SDG target 12.3, which states that “by 2030, halve global per capita food waste at the retail and consumer level and food losses in production chains and supply, including post-harvest losses” should be reduced (FAO, 2015). Additionally, reducing food waste contributes to the achievement of several other SDG, such as UN-SDG 1: End poverty in all its forms everywhere, and UN-SDG 2: End hunger, achieve food security and improved nutrition, and promote sustainable agriculture. According to the United Nations (UN., 2020b), from 2017 to 2019, 79 countries and the European Union reported on at least one national policy instrument that contributed to the implementation of the 10-Year Framework of Programmes on Sustainable Consumption and Production. Regions such as East Asia, Latin America and the Caribbean, Europe, and North America demonstrate a higher level of sustainability reporting, including water use and waste, than Africa, Central Asia, and Oceania (UN., 2020a).

The global food waste problem has drawn strong focus on research and public policy actions, since it is perceived as a phenomenon with negative effects at different levels, such as (i) the loss of money for both the households and the food value chains (e.g., an unnecessary expenditure of $ 750 billion in 2007; FAO, 2013), (ii) the loss of natural resources, which translates into social costs since food is lost while many people starve (e.g., restricted food security in developing countries; Buchner et al., 2012), and (iii) additional greenhouse gas emissions from the agriculture and livestock sector as well as the involved value chain links (e.g., the global carbon footprint of food waste in 2007 was equivalent to 3.3 gigatons CO_2_ emissions; FAO, 2013).

Food waste rather results from the lack of routines and planning in terms of production, purchase, and consumption than from intentional rational processes (Stefan et al., 2013), since most people share the idea of not wasting food (Bolton and Alba, 2012). According to the Food and Agriculture Organization of the United Nations (FAO), food waste, at the consumer level, is frequently associated with (i) non-planning regarding food purchase, (ii) overbought, overly large portions and package sizes, and confusion over labels, and (iii) storage problems at home (FAO, 2019).

Since the global spread of COVID-19 in 2020 and its declaration as a pandemic (WHO., 2020), the world is facing one of the worst challenges in modern times, and the high contagiousness of the virus has forced governments and local authorities to apply measures that help controlling it and protect citizens, including e.g., travel bans, temporary closures of public/private establishments, quarantine/confinement of individuals, and nationwide lockdowns, which have caused significant economic downturns. Although the world had faced previous economic recessions, the COVID-19 pandemic and the associated mitigation measures cause changes in employment, income, and health for the entire population (Rodgers et al., 2021). Angelucci et al. (2020) describe that these measures caused disproportionate job losses for those who could not carry out their activities remotely. Nicola et al. (2020) mention that the pandemic, until the publication of their study, has caused around 141 million confirmed cases and more than 3 million deaths at the global level, leading to fears of an imminent economic crisis only comparable with the economic scenarios during World War II and the Crisis of 1929 (World Bank., 2020). Social distancing, mobility restrictions, job losses, panic buying behavior and product shortages in supermarkets, and the overall transformation from what we considered to be normal to a new normality is now at the center of the daily news.

Not only does the pandemic affect public health, employment, or household incomes, but also the food system and its associated value chains, and disruptions are already noticeable and will likely grow over time (FAO, 2020). Ellison and Kalaitzandonakes (2020) mention that the COVID-19 lockdowns caused that some food did not reach the final consumers and is wasted in the different links of the food value chains.

Regarding the food consumers and their households, there are changes in behaviors regarding food waste, which can be grouped into two types (Ellison and Kalaitzandonakes, 2020; Pappalardo et al., 2020; Wang and Hao, 2020): First, panic caused hoarding effects among food consumers and led to food storage in large quantities, i.e., during the first weeks of confinement, since this gave them a sense of security, increasing household food waste considerably. Second, many households perceive income reductions, leading to less food waste since income and food waste are positively related.

There is no single food waste behavior in the face of the pandemic and its mitigation measures, but a heterogeneity in the behaviors linked to household income (Vidal-Mones et al., 2021). In the United Kingdom, for example, a study by Quinn (2020) revealed that the confinement led to considerable reductions in food waste of up to one third when compared to pre-lockdown levels, i.e., in staple foods, such as potatoes, bread, chicken and milk, which is related to the reduced amount of food consumed outside and the higher consumption of homemade food. Amicarelli and Bux (2020), using the food diary methodology, observed food waste trends during the COVID-19 lockdowns in Italy, and describe that they have resulted in an unprecedented situation that changed all the behavior patterns of individuals, including behavior toward food, and that there was a decrease in food waste resulting from the confinement which forced people to stay at home and gave them more time to learn or improve food planning and storage. Rodgers et al. (2021), through a logistic regression model, evaluated the effect of the lockdowns on decision making regarding food consumption and waste in the United States and Italy, and found that confinements decreased domestic food waste because of the general panic COVID-19 caused, which forced individuals to rethink their supply dynamics to reduce outflows of provisions. In this sense, individuals replaced the consumption of perishable food, such as fruits and vegetables, with prepared foods that last longer without deteriorating and are more easily storable.

The economic crisis caused by COVID-19 can become the worst economic crisis in the Latin Americas history. Unlike in Europe, in Latin American countries, the number of people who obey the instruction to stay at home is smaller, since their economic conditions in many cases prohibits such actions, i.e., the lack of unemployment insurances and formal paid and sick leaves. Additionally, many people live from their incomes as day laborers and have insufficient financial means to buy and store food for several days. Likewise, the crisis generates high levels of fiscal spending and indebtedness, which in many cases in Latin America are unsustainable. Considering this, strict COVID-19 measures, such as lockdowns and closures of restaurants, seem to respond better in countries with solid economic models (i.e., Europe), but can have high social costs in countries such as Latin America (Blofield et al., 2020). Considering the socioeconomic effects of the COVID-19 pandemic in Colombia, decreases in food production, reductions in household consumption, and job losses stand out, and according to Mejía (2020), these effects can lead Colombia to its first recession in the 21st century. In the second semester of 2020, Colombia's Gross Domestic Product had a negative growth rate, a situation that had not happened for around 15 years (UNDP., 2021). In Colombia, ~10 million tons of food are lost and wasted every year, which is equivalent to 34% of the total food supply destined for human consumption—an amount that could nourish more than 8 million people a year, which is equivalent to the population of Colombia's capital Bogotá (DNP, 2016). For Colombia, the proportion of food waste that occurs at the consumption level corresponds to only 15.6% of the total wastage, whereas food loss from agricultural production and post-harvest and storage processes represent 40.5 and 19.8%, respectively, and food loss from distribution and retail 20.6% (DNP, 2016).

Regarding the epidemiological situation in Colombia, by the end of 2020 1,642,775 cases of COVID-19 were reported, with a mortality rate of ~3% (43,213) (Wordometers., 2021). At that time, ~16,300 new cases were reported daily. By September 2021, Colombia was within the Top-10 countries with both confirmed COVID-19 cases (after the United States, Brazil, France, Argentina, among others) and related deaths (after the United States, India, Mexico, among others) (STATISTA., 2021a,b). To get an idea of the severity of the contagion of COVID-19 in Colombia, the Johns Hopkins University and the World Bank conducted an analysis of deaths per 100,000 inhabitants, and Colombia is ranked number nine worldwide, surpassed only by Peru, Hungary, Bosnia and Herzegovina, North Macedonia, the Czech Republic, Bulgaria, Brazil, and Argentina (RTVE., 2021).

The arrival of the outbreak in Colombia cannot be compared with other countries, not even with those from the same region, since in Colombia there are high rates of poverty, unemployment, low quality of life, and non-optimal economic conditions, which is why the arrival of COVID-19 instantly wreaked havoc. Especially when the proposed mitigation measures by the national government are considered, the above-described panorama even got worse since Colombia has such a high share of informal employment and the lockdown and its related economic downturn left behind those who make their living as day laborers. In addition, the pandemic caused stigmatization toward medical personnel, which responds to fear, mistrust and misinformation (Trejos-Herrera et al., 2020). Another effect of the multiple lockdowns and other measures in Colombia was the increase in incidents of domestic violence, depression, suicide, acute and post-traumatic stress, and anxiety, among others (Garfin et al., 2018; Caballero-Domínguez et al., 2020; Duan and Zhu, 2020; Lima et al., 2020; Pedrozo et al., 2020).

The objective of this article is to analyze changes in food waste behavior during the COVID-19 lockdown in Colombia in 2020. Specifically, we focus on two sub-objectives, namely the effects of the lockdown on (i) changes in the *intention* to reduce household food waste, and (ii) the actual *behavior* toward household food waste. We also evaluate whether it is appropriate or not to use the Theory of Planned Behavior (TPB) for predicting the intention and behavior regarding household food waste, based on our results for Colombia as a case study. Data was obtained from a virtual survey conducted during the strict lockdown in July 2020 with 581 households from the four major cities of Colombia: Bogotá, Medellín, Cali, and Barranquilla. With this information and considering the TPB, we apply Random Forest and Ordered Logistic Regression. This article is structured as follows: section 2 postulates the materials and methods, providing information on the theoretical framework, a literature review, data sources, and the description of the different models used. Section 3 presents our findings, section 4 the discussion, and section conclusions and recommendations.

## Materials and Methods

### Theoretical Framework

There is general agreement among social psychologists that most human behavior is goal-directed. Without this being considered frivolously, human behavior is defined by the realization of structured plans (Ajzen, 1985). As Mankiw (2009) postulates within the 10 principles of economics, individuals respond to incentives because they are rational. Ajzen (1985) posits that there is a difference between intentions and actions, and, although all actions are controlled by intentions, generally not all intentions materialize into actions. Considering this, Ajzen (1985) postulates the Theory of Reasoned Action (TRA) in which beliefs, attitudes, and intentions interact to arrive at a certain behavior.

Ajzen (1985) also points out a difference in the degree of volitional consciousness of individuals and considers internal and external factors that can influence motivation when performing difficult tasks. The Theory of Planned Behavior (TPB) is then the result of considering the reasoned action, plus a measure of control over behavior (Ajzen, 1988, 1991). In this sense, in the TPB, intentions can only be expected to predict a person's attempt to perform a behavior, not necessarily their actual performance. The theory considers all the factors beyond a person's control that prevent him from achieving his goal, even if he intends and thus, the immediate determinant of a person's attempt to conduct is his intention to attempt to do so.

The TPB has been validated in multiple research contexts, for example, in studies related to (i) choices on different transportation methods (Bamberg et al., 2003; Gardner and Abraham, 2010) (ii) recycling patterns and conscious consumption (Boldero, 1995; Taylor and Todd, 1995; Sparks et al., 2014), and (iii) predicting patterns of food consumption and food waste (Stefan et al., 2013; Graham-Rowe et al., 2015; Mallinson et al., 2016; Zhang et al., 2019).

In summary, the TPB reflects attitudes toward behavior, the subjective norms, and behavioral control. It is possible to show that there exists a gap between intention and behavior and that both are conditioned by attitudes, subjective norms, and perceived behavioral control. For this article, we use the TPB to analyze household behavior regarding food waste and the questionnaire design was based on Ajzen (1985), contemplating elements such as subjective norms, intentions, attitudes, perceived behavioral control, financial attitudes, identity of a good supplier, and personal identity.

### Literature Review

There is consensus among researchers that people share the idea of not wasting food (e.g., Lyndhurst et al., 2007; Stefan et al., 2013; Jagau and Vyrastekova, 2017). Therefore, it is better to consider that the attitude to waste food is measured as a lack of concern about food waste and not as a right or wrong decision on wasting food (e.g., Lanfranchi et al., 2016). Arvola et al. (2008) and Olsen et al. (2010) considered the influence of moral attitudes on intentions as this improves predictions. In the sense of food waste, people generally feel guilty when they waste or take wasteful attitudes (Bolton and Alba, 2012).

The link of the TPB and food waste was first established by Stefan et al. (2013) to assess if food waste could be related to the food choices and planning within Romanian households. Their results show that moral attitudes toward food waste determine food planning and purchasing routines and, through them, control food waste. In other words, the upright considerations about food waste allow consumers to plan their consumption, hoping they do not have to waste and, therefore, reduce waste.

Graham-Rowe et al. (2015) used the TPB to evaluate fruit and vegetable waste among households in the United Kingdom, applying a linear regression model. They found that attitudes, subjective norms, control of perceived behavior, among others, represented ~64% of the variation of intentions regarding food waste. Regarding changes of behavior, however, they found that only about 5% of the actual behavior changed. As additional predictors to the basic model of planned behavior, they identified consumer identity, early repentance, moral standards, and descriptive norms. Mallinson et al. (2016), also among households in the United Kingdom, identified that household size, packaging format, price awareness, marketing, and behavioral and sociocultural factors influence food waste levels. Stancu et al. (2015) applied the TPB to evaluate the effects of intentions regarding food waste in Denmark, including intention, subjective norms, attitudes toward food waste and perceived behavioral control in their models. They found that intentions, subjective norms, and attitudes contribute to reducing food waste, while perceived behavioral control does not make a significant contribution.

Setti et al. (2018) also postulate the TPB as the indicated option to evaluate aspects related to domestic food waste and based on their study with Italian food consumers suggest that ingrained judgments, values, habits and hunger, the so-called visceral factors (Loewenstein, 1996; Verplanken et al., 1998; Graham-Rowe et al., 2014), influence food decisions, and that there is a gap between food decisions and the results of actual behavior measured as food waste.

Zhang et al. (2019) linked the TPB with consumption behaviors after an outbreak of avian influenza in 2017 in China and found that attitudes, subjective norms, control of perceived behavior, and perceptions about influenza effectively modeled the consumption intention, specifically regarding poultry. This study suggests the suitability of the TPB to model behaviors even when extreme situations like avian influenza or COVID-19 occur.

### Data Sources

To analyze the effects of the COVID-19 lockdown on household food waste behavior, and considering the TPB as theoretical construct, we applied a household survey with food consumers from the four principal cities in Colombia: Bogotá, Medellín, Cali, and Barranquilla. A total of 579 people were surveyed, with an age of minimum 18 years, from the socioeconomic strata 2–6^1^, and with knowledge of their household food expenses and participation in the preparation of food at their homes. The survey was carried out virtually (given the restrictions in Colombia at that time) in July 2020 through an online panel by the company Cluster Research^2^ Below we describe the selected variables and dimensions and their importance.

### Explanatory Variables

Within the explanatory variables, we consider four groups, namely (i) situational predictors of food waste behavior (Visschers et al., 2016), (ii) dimensions postulated by an extended version of the TPB (Carrigan et al., 2006; Evans, 2012; Watson and Meah, 2012; Graham-Rowe et al., 2015; Parizeau et al., 2015; Visschers et al., 2016; McCarthy and Liu, 2017), (iii) the COVID-19 lockdown and the effect of fear and the search for normality, and (iv) sociodemographic control variables.

- Situational predictors of food waste behavior:

- *Household size*: Positively related to the amount of food waste (Koivupuro et al., 2012; Williams et al., 2012; Quested et al., 2013; Parizeau et al., 2015; Schmidt, 2016).
- *Age of the person in charge of food preparation:* The older a person is, the less food waste results (Lyndhurst et al., 2007; Watson and Meah, 2012; Thyberg and Tonjes, 2016).
- *The budget available for food purchase (household income):* The more is spent on food, the higher the food waste is (Watson and Meah, 2012; Quested et al., 2013; Neff et al., 2015; Parizeau et al., 2015).

(ii) Extended version of the TPB:

Regarding the dimensions mentioned by the extended version of the TPB, we constructed the survey considering the basic and most relevant dimensions to make the survey easily accessible for the interviewees. During the data cleaning process, we considered the averages of the items that make up each of the dimensions and constructed eight variables from 22 items, namely (i) *Attitudes about food waste*, (ii) *Subjective norms*, (iii) *Control of perceived behavior*, (iv) *Moral standards*, (v) *Financial attitudes*, (vi) *Identity of a good supplier*, (vii) *Intention to not waste food*, and (viii) *Declared behavior regarding food waste* (Table 1).

**Table 1:** The TPB dimensions considered in this study.

	**On a scale of 1–5, where one means that you disagree and five agree:**				
	**1**	**2**	**3**	**4**	**5**
**Attitudes about food waste (Cronbach's alpha = 0.74**, ***n*** **= 3)**					
I am concerned about the amount of food that is wasted because of its impact on the environment	3.66%	3.14%	10.98%	18.12%	64.11%
I would rather leave food on the plate than eat a lot and gain weight (inverted question)	7.99%	7.12%	14.24%	15.10%	55.50%
I think it is okay to use the leftover foods to prepare other meals	8%	4%	14%	19%	56%
**Subjective norms (Cronbach's alpha = 0.95**, ***n*** **= 3)**					
My household wastes more food than other households of a similar size (inverted question)	4%	3%	6%	19%	68%
People who are important to me think it is good to reduce food waste at home	2%	2%	6%	18%	72%
I think my neighbors and other people close to me are trying to reduce food waste	5%	7%	30%	22%	35%
**Control of perceived behavior (Cronbach's alpha = 0.93**, ***n*** **= 2)**					
I can avoid wasting food during the lockdown	2%	1%	4%	16%	78%
In my household, we manage to avoid food waste	1%	2%	6%	15%	76%
**Moral standards (Cronbach's alpha = 0.80**, ***n*** **= 2)**					
I feel guilty when I throw food away	5%	2%	7%	11%	75%
It is not well seen throwing food away	6%	2%	5%	9%	78%
**Financial attitudes (Cronbach's alpha = 0.73**, ***n*** **= 2)**					
I think throwing away food is a waste of money	7%	4%	7%	13%	69%
I think of the money I lose when I throw away food	7%	4%	11%	16%	62%
**Identity of a good supplier (Cronbach's alpha =0.42**, ***n*** **= 3)**					
I buy fresh produce, although I know not everyone will eat	28%	14%	21%	15%	21%
I like to offer a variety of foods in meals, so that everyone has something they like	11%	7%	21%	22%	39%
I like to buy more food than I need, to have stocks at home	20%	13%	26%	18%	22%
**Intention (Cronbach's alpha = 0.50**, ***n*** **= 2)**					
During the lockdown, I intend to use the remaining food for other meals	6%	5%	14%	17%	58%
During the lockdown, I plan to spend more time planning food purchase to avoid waste	3%	2%	9%	16%	71%
**Declared behavior—food purchase and use (Cronbach's alpha = 0.64**, ***n*** **= 4)**					
During the lockdown, I have given away food that I do not eat	14%	8%	14%	20%	44%
During the lockdown, I prepare and/or keep the remaining food for an upcoming opportunity	8%	6%	13%	18%	56%
During the lockdown, I buy more food than is necessary as a precaution (inverted question)	20%	19%	23%	14%	24%
Before I prepare a meal, I think about how much I need to prepare	2%	1%	5%	16%	77%

*Source: own elaboration*.

(iii) COVID-19 lockdown questions that show the effect of fear and the search for normality:

During extreme situations such as a pandemic, individuals tend to take a self-protective behavior and change their consumption patterns whenever they feel that this will keep them more protected (Lau et al., 2006; Bish and Michie, 2010). The Theory of Protective Action, usually used to describe behaviors in the face of disasters and environmental hazards, postulates that the perceived risk of the situation is the main factor that generates protective behaviors, including behaviors toward consumption (Yeung and Morris, 2001; Pennings et al., 2002; Hornibrook et al., 2005; Lobb et al., 2007; Lindell and Perry, 2012). For this reason, we consider as additional predictors two variables regarding COVID-19 and food consumption:

- *The return to the pre-COVID-19-normality:* How long do you think it will need until the country will return to normality, i.e., the free movement and gathering of persons at anytime and anywhere in the national territory?
- *Concerns regarding the COVID-19 pandemic:* How concerned are you about the COVID-19 situation?

(iv) Sociodemographic control variables:

The behavior toward food waste, whether this is about reduction, reuse, or recycling, responds to personal situations, sociodemographic characteristics, and knowledge/experiences (Barr, 2007). In this sense and following previous studies (e.g., Hines et al., 1987; Barr, 2007; Stancu et al., 2015; Thyberg and Tonjes, 2016) we selected the following sociodemographic control variables:

- *Gender:* There is evidence that women have more pronounced attitudes toward recycling than men (e.g., Barr, 2007; Principato et al., 2015).
- *Educational level:* There is a positive relationship between education and good practices regarding food waste, monthly household income and income reductions due to the COVID-19 pandemic, as households with higher incomes may recognize the importance of not wasting food and have the means not to do so (e.g., Koivupuro et al., 2012; Principato et al., 2015; Setti et al., 2016).
- *City of residence:* There are effects inherent to cities such as cultural aspects (e.g., Stuart, 2009).

**Figure 1:**
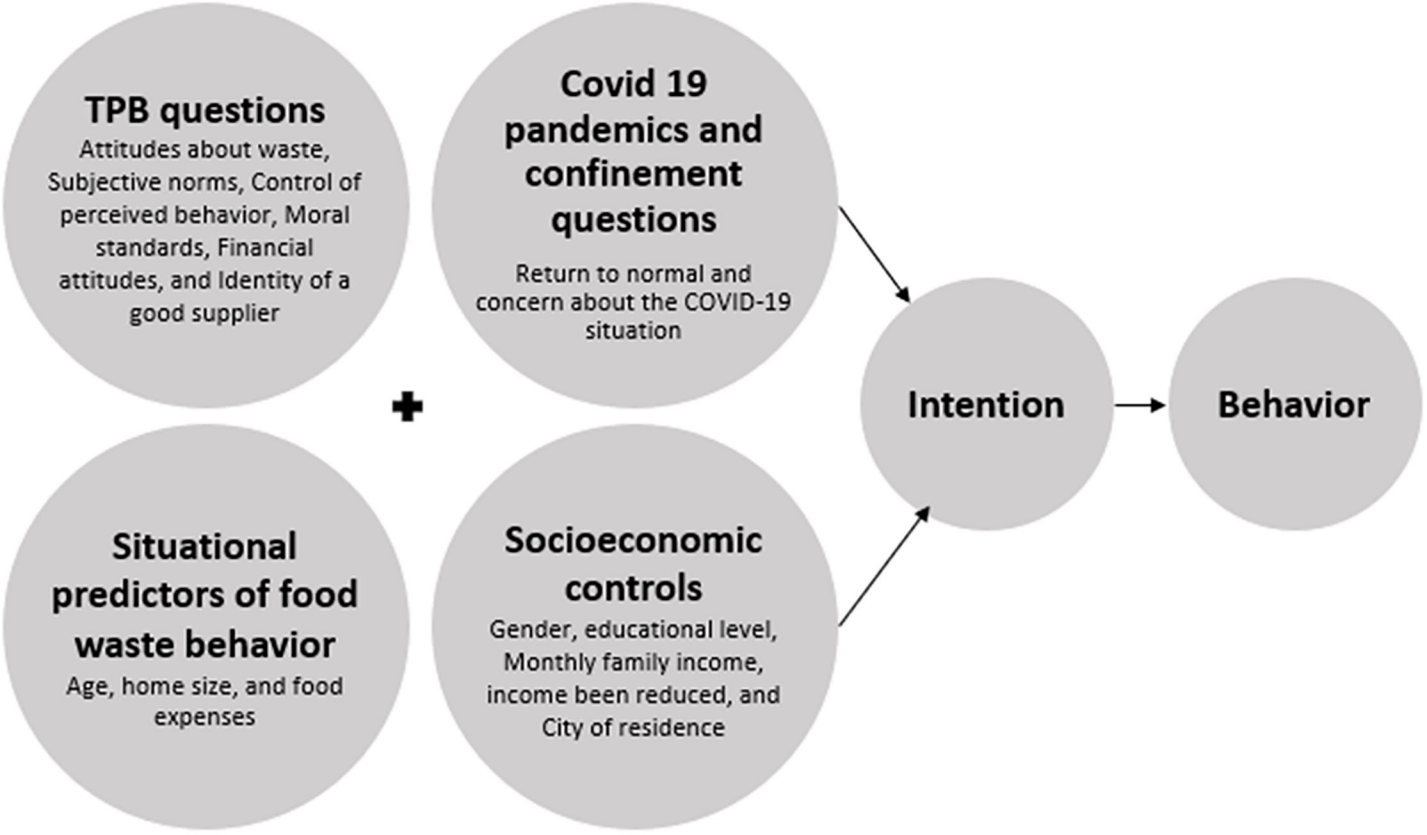
Overview on the dimensions used to predict food waste *intention* and *behavior*. Source: own elaboration.

Figure 1 summarizes the dimensions that were considered in this study, which are consistent with an extended version of the TPB.

### Methodological Approach: Supervised Learning-Random Forest

We consider that supervised learning is a good option to use machine learning since our study counts on a set of labeled data. In supervised learning, a part of the data is used to train the models, so that the algorithm can generalize behaviors and correctly assign classifications. The objective of using supervised learning in this document is to generate models that can classify whether a person would change his behavioral patterns (or intentions) regarding household food waste, given information about their attitudes, subjective norms, and perceived behavioral control, in addition to sociodemographic control variables. The Random Forest joint learning algorithm consists of many decision trees, and, in this sense, it manages to average the classifications/predictions of the individual trees, considerably improving the initial algorithm (Breiman, 2001). One of the desirable characteristics of this algorithm is that it works well regardless of whether the target variable has balancing problems (Breiman, 2001; Liaw and Wiener, 2002).

### Methodological Approach: Ordered and Multinomial Logistic Regression

An ordered logistic regression is an extension of the dichotomous logistic regression model and a regression model for ordinal dependent variables. Considering that the dependent variable related to food waste *intention* is ordinal, we consider this empirical strategy as appropriate.

The model only applies to data that meet the proportional Odds assumption, meaning that the number added to each of these logarithms to get the next is the same in every case. In other words, these logarithms form an arithmetic sequence.

For this article, the scale used is a Likert-scale from 1 to 5, with 5 being the maximum value for each question. We observe that for this set of ordered categories, the behavior of the responses coincides with that of continuous variables, since the increase between the categories occurs by one unit. In this sense, in addition to ordered logistic regression and as a robustness check, we also use a linear regression (considering the variables as continuous) to see the effect of the dependent variables on the *intention* or *declared behavior* regarding food waste. We estimate that the effect of moving from a lower to a higher category coincides with the effect of increasing one unit on the propensity to change. As Grace-Martin (2009) mentions, if the predictor is categorical and fictionally coded, a difference of one unit coincides with the change from one category to another.

An important caveat is that the continuous approach is only being considered as an additional robustness check, since we recognize that this treatment of categories as continuous data may have weaknesses. Thus, we estimate the following equation for *food waste intention*:


(1) $$\begin{array}{l} Intention_{i}=\alpha_{0}+\alpha_{1}SituacionalPredictors+\;\\ \;\alpha_{2}ConfinementQuestions+\alpha_{3}TPB+\;\varepsilon_{i} \end{array}$$

Where *Intention*_*i*_ refers to the average intention rating of individual *i* (1 being the lowest intention and 5 the highest). The *SituacionalPredictors*, *ConfinementQuestions*, and *TPB* are the variables of these categories (see section Explanatory Variables) whose descriptor engineering considers them relevant.

Logistic regression models have many variants in which the dependent variable can have more than two categories (multinomial) or is of ordinal (ordered) character (Long, 1997; Hosmer and Lemeshow, 2000; Peña, 2002; Llaugel and Fernández, 2011). When the dependent variable is the declared behavior of the treatment, it ceases to be ordinal and becomes nominal. In this case, we use a multinomial logistic regression, since the dependent variable responds to independent categories (increased, decreased, and stayed the same). This method corresponds to a generalization of binary logistic regression and is used to model elections, for example. The equation to estimate *food waste behavior* in this case is:


(2) $$\begin{array}{l} Behavior_{i}=\beta_{0}+\beta_{1}SituacionalPredictors+\\ \;\beta_{2}ConfinementQuestions+\beta_{3}TPB+\;\varepsilon_{i} \end{array}$$

Where *Behavior*_*i*_ has two possible presentations: ordinal and multinomial. In the ordinal analysis, *Behavior*_*i*_ refers to the average assessment of individual *i*'s behavior (1 being a negative and 5 a positive assessment). In the multinomial analysis, *Behavior*_*i*_ can take three values (increase, decrease, or stay the same). In this sense, multinomial behavior can be considered a declared behavior while ordinal behavior will only present a potential behavior. Like for Equation (1), The *SituacionalPredictors*, *ConfinementQuestions*, and *TPB* are the variables of these categories (see section Explanatory Variables) whose descriptor engineering considers them relevant.

### Limitations of This Study

The database used in this document has several limitations that must be considered.33ee The most important one is that the measure of household food waste, and all dimensions considered, were self-reported by the survey participants through a virtual survey and collected at a specific point in time (July 2020). Sheen et al. (2020) mention the lack of consensus regarding measures of food waste. Additionally, as mentioned before, the dimensions can respond to prosocial norms with answers that may be biased motivated by the feelings of anguish typical at the beginning of the COVID-19 pandemic (e.g., Quested et al., 2011; Krumpal, 2013). Finally, the sample considered in this study is not necessarily representative as we only obtained data from households in the four major cities of Colombia, which cannot necessarily be generalized for the entire country, since the socioeconomic conditions and cultural habits vary, i.e., among rural areas and cities. For this reason, although the results are relevant and shed light on food waste behavior during the COVID-19 lockdown, which can be used for designing policy responses, the real food waste behavior of the country, might be different.

Additionally, because the responses related to *food waste intention* and *behavior*, among other dimensions of the TPB, are located between the maximum values (4–5) of the scale, the database is unbalanced. This imbalance can bias the results according to the methodological approach used. In this sense, this weakness might be affecting our results (e.g., Geurts et al., 2006; Strobl et al., 2007; Yousefi et al., 2010).

Although logistic regression models work relatively well, they have some weaknesses, namely they can suffer from (i) overfitting, meaning that if the logistic regression is performed on big data sets, the selection of the variable will take a long time (Geng, 2006), and (ii) influence values, which have an undue influence on the overall fit of the model, meaning that the logistic estimates are consistent and asymptotically efficient before a balanced data set only and tend to be biased if there are balancing problems.

In summary, there exist gaps in both approaches (e.g., Colombet et al., 2000; Lee et al., 2005; Steyerberg et al., 2010; Boulesteix et al., 2013), which can be filled by combining them, which is the reason why we decided to implement both algorithms to provide robust results that respond to classical and modern techniques.

## Results

### Descriptive Statistics and First Considerations

Table 2 shows the descriptive statistics of the variables under analysis. We use intention to not waste food and behavior regarding food waste as our main dependent variables. As independent variables, we include sociodemographic control variables in the econometric models.

**Table 2:** Summary statistics of the variables used for the analysis.

**Situational predictors of food waste behavior**	***n***	**%**
**Age**		
18–25 years	107	18.64%
26–35 years	209	36.41%
36–45 years	151	26.31%
46–55 years	92	16.03%
>55 years	15	2.61%
**Household size**		
1 person	15	2.61%
2 persons	89	15.51%
3 persons	157	27.35%
4 persons	195	33.97%
>4 persons	118	20.56
**Have the food expenses in your household changed during the**		
**COVID-19 lockdown?**		
Yes	479	83.45%
No	94	16.38%
NS/NR	1	0.17%
**COVID-19 pandemic and lockdown variables**	***n***	**%**
**How long do you think it will need until the country will return to**		
**normality, i.e., the free movement and gathering of persons at**		
**anytime and anywhere in the national territory?**		
0–3 months	24	4.18%
4–6 months	117	20.38%
7–12 months	207	36.06%
13–18 months	123	21.43%
19–24 months	61	10.63%
>24 months	42	7.32%
**How concerned are you about the COVID-19 situation?**		
Not concerned at all	11	1.92%
A little concerned	65	11.32%
Concerned	278	48.43%
Very concerned	220	38.33%
**TPB dimensions**	**Mean**	**Std**.
Food waste attitudes	4.196181	0.7676408
Subjective norms	4.282986	0.7376499
Control of perceived behavior	4.739583	0.6254564
Moral standards	4.585069	0.8745996
Financial attitudes	4.388889	0.9752493
Identity of a good supplier	3.215278	0.9827252
Intention	4.449653	0.8049196
Declared behavior—food purchase and use	3.998264	0.7138363
**Declared Behavior—total food waste**		
Increased	84	14.63%
Decreased	314	54.70%
Remained the same	176	30.66%
**Sociodemographic control variables**	***n***	**%**
**Gender**		
Male	250	43.55%
Female	324	56.45%
**Educational level**		
High school	94	16.38%
Technician	146	25.44%
Undergraduate	254	44.25%
Postgraduate	80	13.94%
**Monthly household income (in US** ^a^ **)**		
<§291.3	99	17.25%
§291.3–§582.7	169	29.44%
§582.7–§874	128	22.30%
§874–§1,165.3	94	16.38%
>§1,165.3	60	10.45%
NS/NR	24	4.18%
**Reduction of household income during the COVID-19 lockdown?**		
No reduction	94	16.38%
<25%	138	24.04%
25–50%	247	43.03%
51–75%	76	13.24%
76–100%	19	3.31%
**City of residence**		
Bogotá	224	39.02%
Medellín	113	19.69%
Barranquilla	120	20.91%
Cali	117	20.38%

*Source: own elaboration*.

a *The closing US§ exchange rate for 2020 was §3,432.50 Colombian Pesos (COP) Banco de la República. (2021)*.

We observe that ~84% of the surveyed households perceived a reduction in their incomes, and data shows that *behavior* has a strong tendency toward decreasing food waste. Specifically, 55% of the households declared to have reduced food waste, and for 31 and 14%, food waste remained at the pre-pandemic levels or increased, respectively. This finding is preliminary evidence of the positive relationship between *household income* and household food waste during the COVID-19 lockdown.

Furthermore, the logistic regression between the decrease in household food waste and the decrease in *household income* reveals that if the household challenges income decreases, the Odds Ratio of decreasing household waste triples. Specifically, if the household income declines by more than 25%, the Odds Ratio of reducing household food waste ranges between 60 and 75%.

The TPB postulates that there is a gap between *intention* and *behavior*. In this sense, the results are first presented with *intention* as the dependent variable, and then the variable *behavior* is considered.

**Figure 2:**
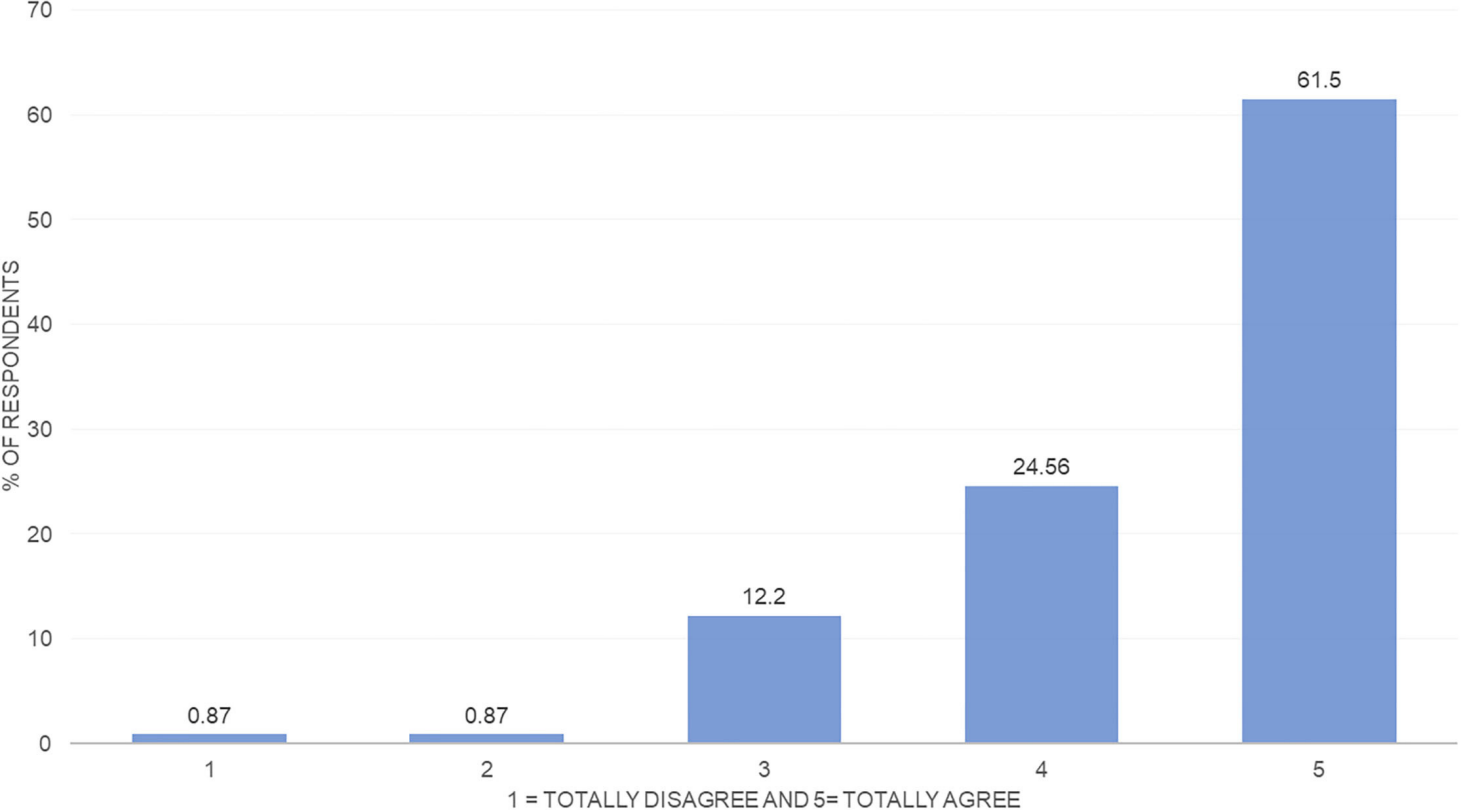
*Intention to not waste food*—distribution. Source: Own elaboration.

**Figure 3:**
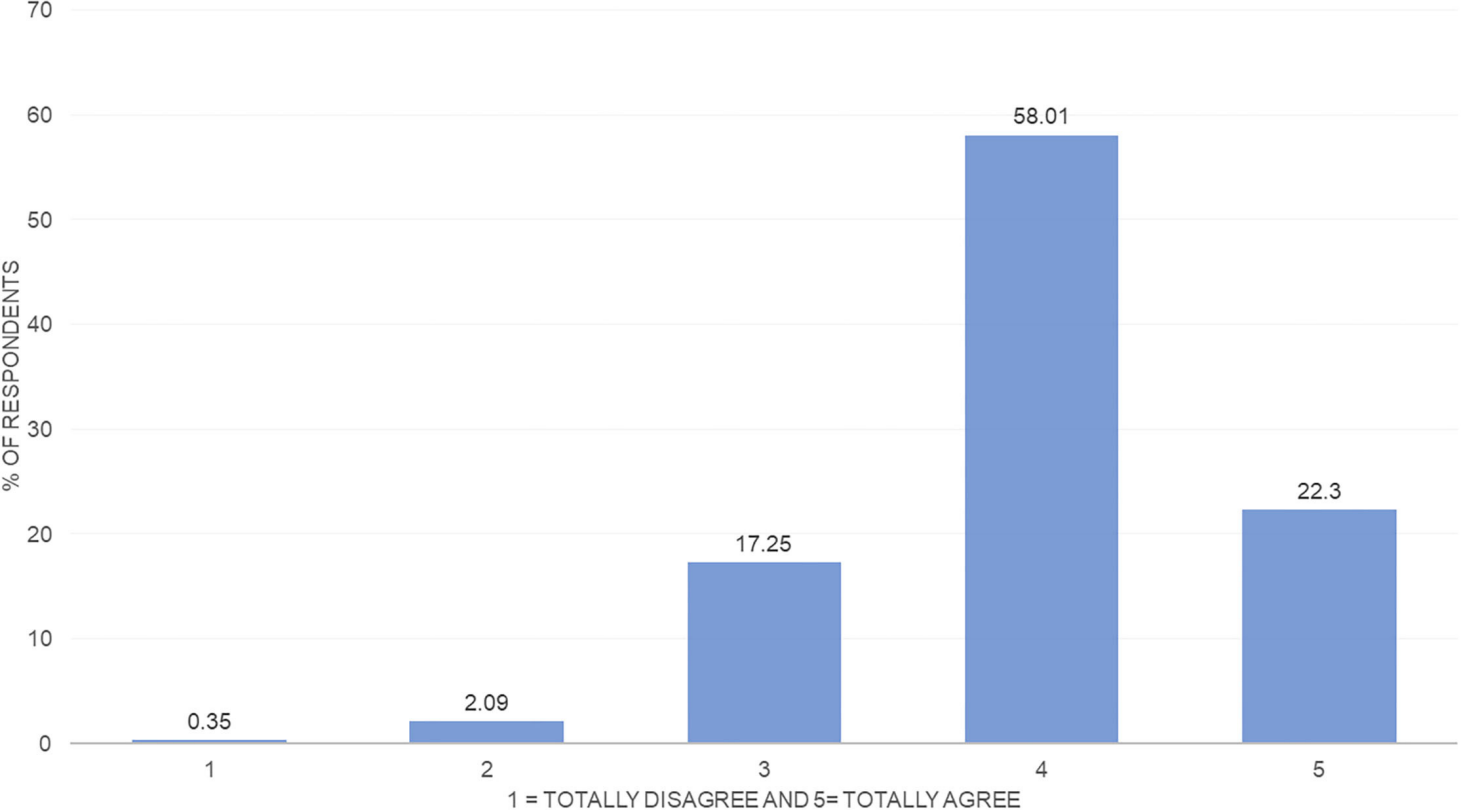
*Declared behavior* regarding food waste—distribution. Source: Own elaboration.

Figure 2 shows that most of the surveyed households agree that they have an *intention to not waste food*. Specifically, 61% fully agree with having an attitude that reduces food waste. Figure 3 shows the distribution of the respondents' *declared behavior*. The comparison of Figures 2, 3 allows to observe the existing gap between *food waste intention* and *behavior*: Although 61% of the respondents fully agree on the *intention to reduce food waste*, only 22% fully agree with having a *behavior* that reduces food waste.

### Consumer Intention to Not Waste Food

For *intention* as dependent variable, the accuracy data are 0.65 for Random Forest, The Random Forest classification model indicates that the total percentage of items classified correctly regarding the *intention to not waste food* is 63.79%. One of the main advantages of this model is that it allows observing the importance of the attributes (independent variables) that compose it, which is the increase in the prediction error if the attribute disappears. This allows us to observe a summary and global vision of the behavior of the model. Table 3 shows the results of the Random Forest model for the variable *intention*.

**Table 3:** Random Forest classification model for the variable *intention to not waste food*.

**Random forest classification model**	
**Test size = 0.2. Dependent variable: intention**	
Food waste attitudes	0.1030
Return to normality	0.1016
Monthly household income	0.0961
Reduction in household income during COVID-19 lockdown	0.0917
Financial attitudes	0.0908
Identity of a good supplier	0.0818
Control of perceived behavior	0.0804
Educational level	0.0795
Subjective norms	0.0723
Concerns about the COVID-19 situation	0.0699
Moral standards	0.0603
Gender	0.0417
Change in food expenses	0.0308
Accuracy	0.6379

*Source: own elaboration*.

The results show that the most significant variables in the classification for the variable *intention to not waste food* are *food waste attitudes, the return to normality (no lockdown), household income, reduction in household income, financial attitudes*, and *control of perceived behavior*. This indicates in a first step that the TPB is a valid framework for our analysis.

We implemented an ordered logistic regression (see robustness check with linear regression in Annex 1), using the most relevant independent variables to strengthen our analysis. The variables selected for the variable *intention to not waste food* are *return to normality* (*no lockdown*), *household income, food waste attitudes, reduction in household income, financial attitudes, control of perceived behavior, educational level, concerns about the COVID-19 situation, subjective norms, gender, food expenses, moral standards*, and *identity of a good supplier*.

The results of the ordered logistic regression (Table 4) suggest that only the variables *concern about the COVID-19 situation, food waste attitudes, financial attitudes, subjective norms, and control of perceived behavior* are significant in explaining changes in the *intention regarding food waste*. We observe that in both the Random Forest model and the logistic models, the variables related to the TPB effectively explain changes in the *intention to not waste food*. The logistic regression uses Odds Ratio as standardized measure to compare the effect of the independent variables on the dependent variable. Thus, an increase in the *food waste attitude* (which means that a person is more in agreement with not wasting food) more than doubles (2.2896) the Odds Ratio that the *intention to not waste food* increases, keeping all other variables constant. Likewise, an increase in the *control of perceived behavior* (which means that a person feels that he has more control over the level of waste) increases the Odds Ratio by 1.8649 that the *intention to not waste food* will increase.

**Table 4:** Ordered logistic regression for the variable *intention to not waste food*.

**Ordered logistic regression. Dependent variable: Intention**		
**Variables**	**Intention**	**Odds ratio**
Reduction in household income during COVID-19 lockdown	n.a.	
Return to normality	n.a.	
Concerns about the COVID-19 situation	0.331**	1.3244
	(0.141)	
Food waste attitudes	0.814***	2.2896
	(0.151)	
Financial attitudes	0.489***	1.6644
	(0.102)	
Moral standards	n.a.	
Identity of a good supplier	n.a.	
Subjective norms	0.370**	1.4035
	(0.151)	
Control of perceived behavior	0.590***	1.8649
	(0.199)	
Food expenses	n.a.	
Gender	n.a.	
Educational level	n.a.	
Monthly household Income	n.a.	
cut1	5.074***	
	(1.306)	
cut2	6.196***	
	(1.172)	
cut3	9.119***	
	(1.228)	
cut4	10.888***	
	(1.275)	
Observations	576	
*n*	576	

*Source: own elaboration*.

*Robust standard errors in parentheses*.

*** *p < 0.01*,

** *p < 0.05*.

*n.a., variables that are not significant at any level of significance*.

The cut points shown at the bottom of the output indicate where the dependent variable is cut to form the five groups we observed in our data, having in mind that this latent variable is continuous.

### Behavior Regarding Food Waste

In a first step, we evaluate *behavior* under a Likert scale in which the survey participants had to define from 1 to 5 how much they agree with the following statements: (i) I have given away food that I do not eat, (ii) I prepare and/or keep the remaining food for an upcoming opportunity, (iii) I buy more food than necessary as a precaution (inverted question), and (iv) Before I prepare the meal, I think about how much I need to prepare. In this sense, *behavior* is evaluated against many fronts that reduce household food waste, but not against the actual reduction of food waste. In a second step, we evaluate the *declared behavior* against the *actual reduction of household food waste*.

#### Potential Behavior

Using *potential behavior* as dependent variable, the accuracy data are 0.48 for Random Forest, Table 5 shows the results of the Random Forest model for the variable *ordinal declared behavior*.

**Table 5:** Random Forest classification model for the variable *ordinal declared behavior*.

**Random forest classification model**.	
**Test size = 0.2. Dependent variable: Declared behavior**	
Intention	0.291716
Financial attitudes	0.287328
Control of perceived behavior	0.273274
Gender	0.147683
Accuracy	0.4828

*Source: own elaboration*.

This finding suggests that the TPB framework responds well to analyzing the decision to reduce household food waste during the COVID-19 lockdown. It also shows *that financial attitudes* play a significant role, highlighting once again that there exists a relationship between *household income* and *behavior toward food waste*.

Additionally, we performed an ordered logistic regression (see robustness check in Annex 2) to check our results (Table 6). The variables selected for *behavior* are *financial attitudes, intention, control of perceived behavior*, and *gender*.

**Table 6:** Ordered logistic regression for the variable *potential behavior*.

**Ordered logistic regression. Dependent variable: declared behavior**		
**Variables**	**Declared behavior**	**Odds ratio**
Financial attitudes	n.a.	
Intention	0.908***	2.479412
	(0.154)	
Control of perceived behavior	0.723***	2.060147
	(0.157)	
Gender		
cut1	1.508	
	(0.972)	
cut2	4.112***	
	(0.867)	
cut3	6.884***	
	(0.868)	
cut4	10.009***	
	(0.932)	
Observations	576	
*n*	576	

*Source: own elaboration*.

*Robust standard errors in parentheses*.

*** *p < 0.01*.

*n.a., variables that are not significant at any level of significance*.

For the variable *declared behavior*, the logistic regression shows that only the variables *intention* and *control of perceived behavior* are significant, which coincides with what is established by the TPB. An increase by one unit in the variable *intention* (which means that a person has a higher intention to not waste food) increases the Odds Ratio by 2.5 that the *declared behavior* increases. In the same way, if the *control of perceived behavior* increases by one unit, the Odds Ratio of the *declared behavior* increases by 2.1.

#### Declared Behavior

Using *declared behavior* as dependent variable, the accuracy data are 0.47 for Random Forest, Table 7 shows the results of the Random Forest model for the variable *nominal declared behavior*.

**Table 7:** Random Forest classification model for the variable *declared behavior*.

**Random forest classification model**	
**Test size = 0.2. Dependent variable: Declared Behavior**	
Financial attitudes	0.382862
Intention	0.263455
Control of perceived behavior	0.228492
Gender	0.125191
Accuracy	0.4741

*Source: own elaboration*.

Like for *declared behavior*, this finding suggests that the TPB framework responds well to analyzing the decision to reduce household food waste during the COVID-19 lockdown, and that *financial attitudes* play a significant role, highlighting again that there is a relationship between *household income* and *behavior toward food waste*.

Additionally, we performed an ordered logistic regression to check the results. The variables selected for *behavior* are *financial attitudes, intention, control of perceived behavior, gender, reduction in household income*, and *changes in food expenses*.

For *declared behavior*, the multinomial logistic regression (Table 8) shows that only the variables *reduction in household income* and *changes in food expenses* are significant, revealing an incompatibility with what is established by the TPB. The reason for this is that the COVID-19 pandemic and the resulting mitigation strategies (such as lockdowns) caused one of the most serious economic crises in recent years, which led to reductions in *household incomes*, and, although the TPB manages to predict the *intention to not waste food*, when it comes to materializing this *intention*, the effects of the economic variables (*reduction in household income* and *changes in food expenses*) overshadow the effects of other variables.

**Table 8:** Multinomial logistic regression for the variable *declared behavior*.

**Multinomial logistic regression. Dependent variable: Declared Behavior**			
**Variables**	**Increased**	**Decreased**	**Base line (Stayed the same)**
Percentage reduction in household income (1)	n.a.	0.805**	
		(0.313)	
Percentage reduction in household income (2)	n.a.	1.076***	
		(0.291)	
Percentage reduction in household income (3)	n.a.	1.590***	
		(0.405)	
Percentage reduction in household income (4)	n.a.	1.473**	
		(0.644)	
Changes in household food expenses	1.078***	n.a.	
	(0.408)		
Gender	n.a.	n.a.	
Intention	n.a.	n.a.	
Control of perceived behavior	n.a.	n.a.	
Financial attitudes	n.a.	n.a.	
Observations	575	575	575
*n*	575	575	575

*Source: own elaboration*.

*Robust standard errors in parentheses*.

*** *p < 0.01*,

** *p < 0.05*.

*n.a., variables that are not significant at any level of significance*.

## Discussion

### Household Food Waste During the COVID-19 Pandemic

The results of our study are in line with other research on food waste. Although the TPB explains the variable *intention to not waste food* to be important for predicting *declared behavior* or *actual behavior*, other variables, such as *socioeconomic controls*, become relevant, too, supporting the findings of Conner and Armitage (1998). As mentioned by Stefan et al. (2013) and Graham-Rowe et al. (2015), the *intention to reduce household food waste* can be predicted by considering the variables *attitudes, subjective norms*, and *control of perceived behavior*.

Regarding *declared behavior*, the *intention not to waste food* has no significant effects on the variable *nominal declared behavior* since pandemic-related economic factors overshadow the effects of other factors, which coincides with Stefan et al. (2013) who described that although the planning and purchasing routines manage to explain food waste behavior, the *intention* seems not to be significant. This result reinforces the existing gap between *intention* and *behavior* (e.g., Armitage and Conner, 2001; Sheeran, 2002). Graham-Rowe et al. (2015) found that the variation of intentions with respect to food waste explained by the dimensions of the TPB is ~64%; however, with respect to behavioral changes, they found that only about 5% of the actual behavior changed.

The circumstances of the COVID-19 pandemic must be considered within the predictions of *intention* and subsequently of *behavior* (as ordinal and nominal variable), which is in line with the findings of Rodgers et al. (2021), who describe that the pandemic has created circumstances which have resulted in both changes in *attitudes* and *behavior* when it comes to household food waste.

The findings of this paper support the first hypothesis that, according to the TPB model (Ajzen, 1988, 1991), *attitude, subjective norms*, and *perceived behavioral control* can predict the *intention to reduce household food waste*. Households in favor of food waste reduction, feel that other people also approve the reduction of food waste, leading to a higher likelihood of developing an *intention to reduce household waste*, which was also shown by (Arvola et al., 2008; Olsen et al., 2010; Stefan et al., 2013; Stancu et al., 2015). Additionally, we found that *financial attitudes, concerns regarding COVID-19 and the return to normality* (*no lockdown*) are also significant variables for the *intention to reduce household food waste*, which is in line with the study presented by Zhang et al. (2019).

Regarding *ordinal behavior*, it can be predicted considering the variables *intention* and *control of perceived behavior*, which is in line with what was proposed by the TPB. Both predictors double the probabilities of behavioral changes. For *nominal behavior*, the results are, however, not in line with what was proposed by the TPB, since it suggests that only the variables *reduction in household income* and *changes in food expenditures* predict it. As mentioned in the results, we consider that this might be due to the strong impacts the COVID-19 pandemic had on household incomes in Colombia.

As Grace-Martin (2019) mentions, logistic regression is a good tool to have in the statistical tool belt. Within the logistic regressions, the most used for categorical results with more than two categories are the multinomial and ordinal varieties. As they have different assumptions, however, the results are also different (e.g., Pedhazur, 1997; Schwab, 2002). Leadbeater (2019) shows that one of the differences between ordered and multinomial models (in addition to their assumptions) is that the ordinal model appears to be more useful as a generalization and “top view” procedure. Otherwise, multinomial models are more appropriate for a more detailed view of group-level comparisons.

We find it interesting to consider both behaviors (ordinal and nominal), since although the TPB can predict *ordinal behavior*, it seems to fail predicting *nominal behavior*. We emphasize that although there have been more economic crises, the crisis generated by the COVID-19 pandemic had and has an unprecedented effect since it changed each of the activities that defined our daily lives in the pre-pandemic era. This result is consistent with the literature which states that in a crisis in which people feel threatened, their behaviors will be anticipated to take measures to avoid suffering the negative effects (Chapman and Coups, 2006; Leppin and Aro, 2009; Rivis et al., 2009). Furthermore, as mentioned above, the results of the multinomial analysis are more detailed and may account for a more specific effect.

## Conclusions

This document examined the effectiveness of the Theory of Planned Behavior to predict the *intention* and *behavior regarding household food waste* in the four major cities of Colombia, considering the COVID-19 lockdown as a case study. This research seeks to provide robust results regarding strategies that allow reducing household waste considering the consumers' preferences and their attitudes toward external shocks and, therefore, is a contribution to the existing empirical evidence, which allows authorities and decision-makers considering and understanding consumer preferences and developing more effective and efficient policies regarding food waste and responsible production and consumption.

Considering that the COVID-19 pandemic is perhaps the most critical event for humanity in recent history, our literature review revealed that research is being conducted that seeks to assess the economic and social effects of the crisis. Since the pandemic is an unprecedented event, the findings are, however, ambiguous. This study, by using modern and conventional techniques of analysis, therefore contributes to the literature by evaluating the effects of COVID-19 on an issue as intrinsic as food waste and consumption patterns and seeks to provide a point of comparison for future research.

The results found in this study respond to a specific point of time and may vary as personal values, preferences, and the information available about COVID-19 change. The results provide, however, information that shows the suitability of the Theory of Planned Behavior for analyzing food waste intentions and behavior, even when considering a critical event of shock for the households such as the COVID-19 lockdown.

Our results allow for providing policy recommendations that achieve behavioral modifications to reduce food waste at home. Although personal considerations about household food waste (i.e., intention, attitudes, and some other TPB dimensions) are relevant predictors, the differential factor between the willingness to not to waste food and effectively not doing it lies in the economic conditions of the households. This highlights the need for strategies aimed at reducing household food waste that consider the different existing income and food expenditure levels, and, above all, the reductions in household incomes caused by the COVID-19 pandemic (Ruiz-Roso et al., 2020; Vidal-Mones et al., 2021). Additionally, fear and concern about are also significant predictors (e.g., Lobb et al., 2007; Stefani et al., 2008), highlighting the need of accurate communication strategies regarding the COVID-19 pandemic and its impacts on society, e.g., by providing correct numbers on infections, unemployment, deaths, and mitigation measures, for reducing panic-driven consumption behaviors.

## Data Availability Statement

The raw data supporting the conclusions of this article will be made available by the authors, without undue reservation.

## Ethics Statement

The studies involving human participants were reviewed and approved by Internal Review Board of the Alliance of Bioversity International and CIAT. The patients/participants provided their written informed consent to participate in this study.

## Author Contributions

AC, KE, MD, and SB: conceptualization and resources. DM, AC, and MD: methodology. DM: formal analysis. DM, MD, and SB: writing the original draft and review and editing. SB: supervision, funding acquisition, and project administration. All authors contributed to the article and approved the submitted version.

## Funding

This work was undertaken as part of the CGIAR Research Program (CRP) on Livestock.

## Conflict of Interest

The authors declare that the research was conducted in the absence of any commercial or financial relationships that could be construed as a potential conflict of interest.

## Publisher's Note

All claims expressed in this article are solely those of the authors and do not necessarily represent those of their affiliated organizations, or those of the publisher, the editors and the reviewers. Any product that may be evaluated in this article, or claim that may be made by its manufacturer, is not guaranteed or endorsed by the publisher.

## Footnotes

^1^Socioeconomic stratification in Colombia categorizes housing units in a scale of one to six according to their physical characteristics, immediate surroundings and rural or urban context. Higher strata (five and six) and lower strata (one, two and three) [Departamento Administrativo Nacional de Estadística (DANE), 2021]. Retrieved from: https://www.dane.gov.co/index.php/servicios-al-ciudadano/servicios-informacion/estratificacion-socioeconomica

^2^ https://clusterresearch.com.co/

## ANNEX

**TABLE A1:** Linear regression for the variable *intention to not waste food* (robustness check).

**Linear regression. Dependent variable: intention**	
**Variables**	**Intention**
Reduction in household income due to COVID-19 lockdown	
Return to normality	
Concerns about the COVID-19 situation	0.0787*
	(0.047)
Food waste attitudes	0.2601***
	(0.040)
Moral Standards	
Financial attitudes	0.164***
	(0.033)
Personal identity	0.073*
	(0.042)
Control of perceived behavior	0.292***
	(0.078)
Gender	
Educational level	
Monthly household Income (1–4)	
Monthly household Income (5)	0.195*
	(0.118)
**Constant**	
Observations	576
*R*-squared	0.3138
*N*	576

*Robust standard errors in parentheses*.

*** *p < 0.01, and*

* *p < 0.1*.

**Table A2:** Linear regression for the variable *declared behavior* (robustness check).

**Linear regression. Dependent variable: declared behavior**	
**Variables**	**Declared behavior**
Financial attitudes	
Intention	0.287***
	(0.048)
Control of perceived behavior	0.240***
	(0.051)
Gender	
Constant	1.275***
	(0.257)
Observations	576
R-squared	0.231
*N*	576

*Robust standard errors in parentheses*.

*** *p < 0.01*.
